# 

**Published:** 1888-04-14

**Authors:** 


CONTENTS.

The Obligations of Knowledge - 17

Words of Consolation. Chapter LXXIII. - 18

Voluntary versus State-Supported Hospitals. By the Right Hon. the Earl of ROSEBERY, K.T. - 19

The Doctor's Pulpit.—Nerve-tides - 21

Physiology and its Uses - 22

Facts About the London Hospital - 23

Round About the Asylums.—IV. - 23

Notes and News - 24

Everybody's Page - 30

Annotations.—A Medical Prodigy at Glasgow.—An Interesting Centenary at York.—Are Midwives Despised? - 31

Comparative Expenditure of Hospitals - 32

An Ishmaelite. By LESLIE KEITH, Author of "The Chilcotes," "East and West," etc. - 33

Turning over New Leaves - 35

Amusements with Profit for all Ages.—For Men and Women.—Children's Corner.—Puzzles, etc. - 36

EXTRA SUPPLEMENT.—THE NURSING MIRROR.

En Passant - 25

The Registration of Trained Nurses - 26

Lectures - 27

Fresh Fields - 27

Anecdotes of Personal Adventure - 28

Notes and Queries - 28

Appointments—Vacancies - 28

Exchange and Mart - 28

